# Histoire d’un itinéraire épidémiologique entre le Burkina Faso et la Côte d’Ivoire : le cas des foyers de maladie du sommeil de Koudougou

**DOI:** 10.1051/parasite/2012194397

**Published:** 2012-11-15

**Authors:** D. Kiendrébéogo, R. Kambiré, V. Jamonneau, K. Lingué, P. Solano, F. Courtin

**Affiliations:** 1 Institut de Recherche pour le Développement (IRD), UMR IRDCIRAD 177 INTERTRYP, Centre International de Recherche-Développement pour l’Élevage en zone Sub-humide (CIRDES) n° 559 rue 5-31 angle avenue du Gouverneur Louveau 01 BP 454 Bobo-Dioulasso 01 Burkina Faso; 2 Programme National de Lutte contre la Trypanosomose Humaine Africaine 03 BP 7009 Ouagadougou Burkina Faso; 3 Programme National d’Élimination de la Trypanosomose Humaine Africaine 17 BP 934 Abidjan Côte d’Ivoire

**Keywords:** maladie du sommeil, géographie, histoire, propagation, diffusion, Côte d’Ivoire, Burkina Faso, sleeping sickness, geography, history, spread, diffusion, Ivory Coast, Burkina Faso

## Abstract

Dans la première moitié du XXème siècle, alors que la Haute-Volta (actuel Burkina Faso) subissait une terrible épidémie de maladie du sommeil, l’administration coloniale française a orchestré des déplacements massifs de populations de la Haute-Volta vers la Côte d’Ivoire, pour exploiter le territoire. Cela a conduit à la mise en place de villages de colonisation Mossi en zone forestière ivoirienne, comme ceux de Koudougou, issus de l’une des régions les plus peuplées de Haute-Volta, mais aussi l’une des plus touchées par la maladie du sommeil. Depuis 2000, au Burkina Faso, c’est dans le district sanitaire de Koudougou que sont dépistés passivement le plus grand nombre de trypanosomés en provenance de Côte d’Ivoire. Qui sont-ils ? Où habitent-ils au Burkina Faso ? D’où viennent-ils de Côte d’Ivoire ? Après avoir retracé l’histoire épidémiologique des villages de Koudougou au Burkina Faso et en Côte d’Ivoire, nous avons recherché les trypanosomés dépistés passivement depuis 2000 dans le district sanitaire de Koudougou au Burkina Faso. Au total, dix trypanosomés ont été enquêtés. Le processus de propagation de la maladie du sommeil dans l’espace ivoiro-burkinabé a été mis en évidence et des zones à risque de la maladie identifiées dans ce même espace.

## Abstract (en)

In the first half of the XXth century, while Upper-Volta (now Burkina Faso) was suffering a terrible epidemic of sleeping sickness, the French colonial administration encouraged the movement of people from Upper-Volta to Ivory Coast to meet their demands for labour. This led to the establishment of Mossi villages, such as those of Koudougou, in the Ivorian forest with populations originating from areas of Upper-Volta that were not only densely populated but also severely affected by sleeping sickness. Since 2000, most cases of sleeping sickness in the Koudougou district of Burkina Faso have been in people originally from Ivory Coast. Who are they? Where did they settle in Burkina Faso? Where do they come from in Ivory Coast? After having retraced the epidemiological history of Koudougou villages in Burkina Faso and Ivory Coast, the history of ten cases of sleeping sickness detected passively at Koudougou hospital since 2000 were analysed. All cases originated from the forest area of Ivory Coast. Understanding the spread of sleeping sickness between Burkina Faso and Ivory Coast will assist in the identification of areas of disease risk.

## Introduction

Dans la première moitié du XXème siècle, une partie de la population voltaïque (ex Haut- Sénégal-Niger, ex-Haute-Volta, ex-Haute Côte d’Ivoire, actuel Burkina Faso) a été déplacée vers la zone forestière ivoirienne par l’administration coloniale française pour l’aménagement (routes, bâtiments administratifs, chemin de fer, wharfs) et l’exploitation du territoire (développement des cultures de café et de cacao notamment) (Bi Zan, 1973). À cette époque en Afrique Occidentale Française (AOF), le Burkina Faso était le pays le plus touché par la Trypanosomiase Humaine Africaine (THA ou maladie du sommeil), une pathologie due à un trypanosome transmis à l’homme par la piqûre d’une glossine ou mouche tsétsé (Gouzien, 1908). En effet, sur un total de 45 000 trypanosomés dépistés de 1931 à 1934 en AOF, plus de 30 000 provenaient du Burkina Faso (Jamot, 1933). Une partie de la population voltaïque, dirigée vers la zone forestière ivoirienne à cette époque, est donc arrivée trypanosomée, mais avec des taux d’infection variables selon la région d’origine burkinabé, les régions les plus touchées étant le Centre (Mossi) et le Sud-Ouest (Lobi, Bobo) du pays (Muraz, 1943).

L’une des stratégies préconisées par l’administration coloniale française pour implanter les populations en zone forestière ivoirienne a été la création en 1936 (au temps fort de l’épidémie au Burkina Faso) de villages de colonisation (Koudougou, Ouagadougou, Kaya, Tenkodogo, Koupéla, Garango). Ces villages, peuplés essentiellement par des voltaïques, ont été mis en place dans le but précis de développer des plantations de café et de cacao dans le Centre-Ouest ivoirien (Mandé, 1997). C’est principalement à partir de ces villages de colonisation qui vont constituer un point d’attache familial pour les populations restées au Burkina Faso, que les populations burkinabées originaires de ces régions vont s’étendre dans les différentes zones forestières de Côte d’Ivoire (Daloa, Vavoua, Sinfra, Bonon, Abengourou, Danané, Zoukougbeu, Grand-Zatry, Méadji, San Pedro, etc.), à la recherche d’un lopin de terre pour développer une plantation de café et/ou de cacao (Hervouët et al., 2000). Ces migrants agricoles, dont certains sont contaminés, vont par leur extrême mobilité et le cadre entomologique dans lequel elle va s’effectuer, c’est-à-dire l’expansion du vecteur majeur, la tsé-tsé *Glossina palpalis palpalis*, favoriser la diffusion de la maladie du sommeil en zone forestière ivoirienne (Laveissière & Kiénou, 1979). Ceci d’autant plus que la distribution de ces migrants voltaïques dans une multitude de petits campements situés aux fins fonds de la forêt et très difficiles d’accès, ne permettra pas aux équipes médicales alors en charge de la lutte contre la maladie du sommeil, de stériliser totalement le réservoir de parasites chez ces populations (Hervouët & Laveissière, 1988).

Si le Burkina Faso ne déclare plus de cas autochtone de maladie du sommeil depuis le début des années 1990, des trypanosomés en provenance de Côte d’Ivoire y sont régulièrement dépistés (Millogo *et al.*, 1999 ; Simarro *et al.*, 2010 ; Kambiré *et al.*, 2012). C’est notamment le cas à Koudougou-BF (Burkina Faso) où le nombre de cas dépistés semble avoir augmenté ces dernières années probablement du fait des crises pré- et post-électorales ivoiriennes qui ont provoqué le retour de burkinabés (*observ. pers.*). Cette situation nous a amenés à nous interroger sur la distribution spatiale des trypanosomés dépistés passivement depuis 2000 à Koudougou-BF dans l’espace ivoiro-burkinabé.

Afin de replacer cette problématique dans son contexte, nous avons retracé les processus migratoires qui ont participé au peuplement des villages de colonisation Mossi, et plus particulièrement ceux de Koudougou- CI (Côte d’Ivoire). Cette histoire a été recoupée avec les informations disponibles sur la maladie au niveau des lieux de départ et d’arrivée des migrants, dans le but de clarifier la dynamique spatiale de la maladie du sommeil dans l’espace ivoiro-burkinabé. Une fois que cette revue bibliographique a été complétée, nous nous sommes rendus dans la région de Koudougou-BF afin de retrouver les trypanosomés dépistés passivement à l’hôpital de Koudougou depuis 2000, et les avons questionnés sur leurs lieux de vie au Burkina Faso et en Côte d’Ivoire. Au total, dix trypanosomés ont été retrouvés et enquêtés. La caractérisation de leurs parcours géographiques nous a permis de mieux comprendre les processus de peuplements suivis par les migrants burkinabés des villages de Koudougou- CI, et donc de mieux évaluer leur rôle dans le processus de diffusion de la maladie en zone forestière ivoirienne. L’identification de leurs lieux d’habitations dans l’espace ivoiro-burkinabé a permis de déterminer des peuplements à risque de maladie du sommeil. Ces résultats contribueront à l’orientation géographique des activités de surveillance médicale dans l’espace ivoiro-burkinabé.

## Matériels et Méthodes

### Présentation de la zone d’étude

La ville de Koudougou (“forge” en Mooré), cheflieu de la région du Centre-Ouest, est située au coeur du Burkina Faso sur le plateau Mossi. Cette région couvre une superficie de 21 807 km2 pour une population d’environ 1 900 000 habitants en 2006, soit une densité de 87 habitants/km2. La ville de Koudougou est une capitale régionale qui s’appuie sur une population avoisinant 90 000 habitants. Elle est la troisième ville du Burkina Faso, derrière Ouagadougou (1,5 million) et Bobo-Dioulasso (500 000) (INSD, 2006). La zone est soumise au climat nord-soudanien avec une pluviométrie moyenne annuelle d’environ 800 mm. Le couvert végétal est dominé par une savane arbustive sur les interfluves accompagnée par des forêts-galeries plus ou moins dégradées le long des cours d’eau, en particulier le long du Mouhoun (ex-Volta Noire), principal fleuve situé à 45 kilomètres à l’ouest de Koudougou et orienté nord-sud dans cette région.

C’est dans ces forêts-galeries que vivent les glossines, dont deux espèces capables de transmettre le trypanosome pathogène pour l’homme : *Glossina palpalis gambiensis* et *G. tachinoides*. Dans la zone de Koudougou, où les cours d’eau sont tous des affluents temporaires du Mouhoun bordés d’une végétation arbustive éparse, l’espèce dominante était *G. tachinoides* (Challier & Dedewanou, 1967). Ces dernières années, les glossines étaient toujours présentes le long du fleuve Mouhoun, mais avaient en revanche disparu le long des cours d’eau situés aux alentours de Koudougou, du fait d’une dégradation poussée de la végétation liée à une augmentation des densités de population. Aujourd’hui, avec la lutte entomologique menée par la Pan African Tsetse and Trypanosomosis Eradication Campaign (PATTEC) du Burkina Faso, les glossines ont presque totalement disparu de la boucle du Mouhoun (Dr Sidibé, coordinateur PATTEC Burkina Faso, *com. pers.*).

### Recueil d’informations historiques sur les migrations et la maladie du sommeil

**Figure 1.**
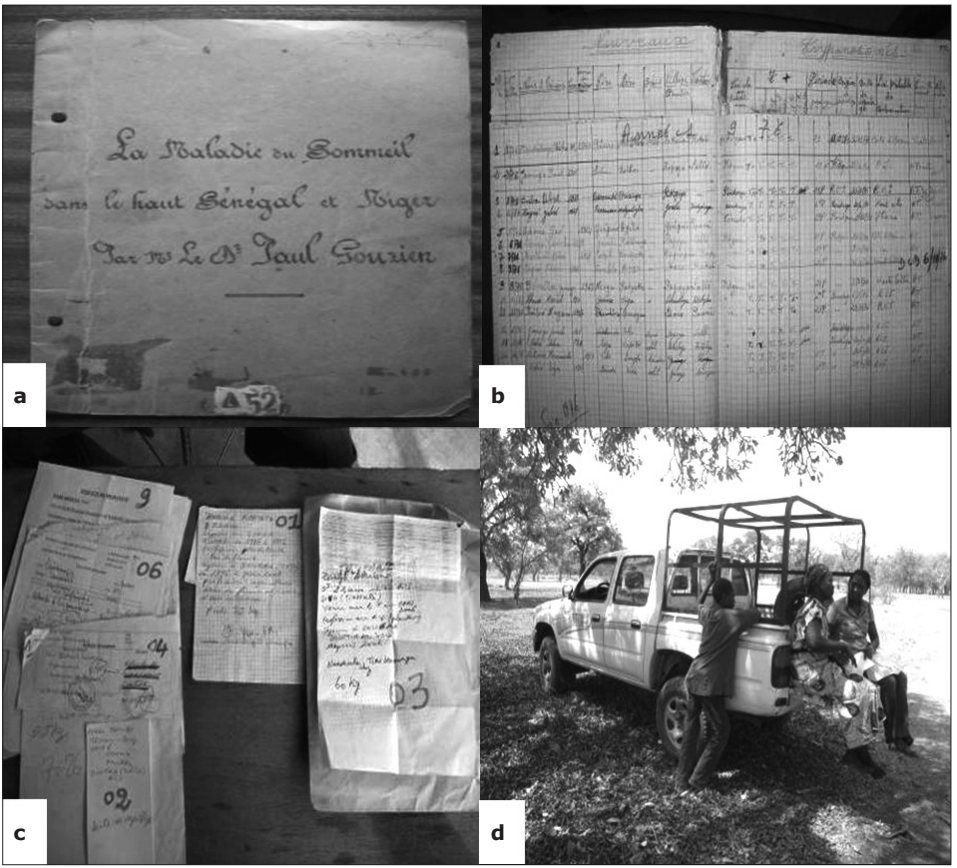
(a) Rapport original de Paul Gouzien (1908) ; (b) une page du registre ; (c) les feuilles individuelles et (d) enquête avec une trypanosomée.

Les fonds documentaires des bibliothèques de différents instituts ont été exploités pour faire le point sur les migrations et la maladie du sommeil dans la région de Koudougou. Concernant les phénomènes migratoires, il s’agit des bibliothèques du Centre d’Études Économiques et Sociales de l’Afrique de l’Ouest (CESAO) à Bobo-Dioulasso, de l’Institut de Recherche pour le Développement (IRD) à Ouagadougou et du Centre d’Archive d’Outre-Mer (CAOM) à Aix-en-Provence. Les documents sur la maladie du sommeil ont été consultés dans les bibliothèques de l’Organisation Ouest Africaine de la Santé (OOAS) à Bobo-Dioulasso où se trouvent les rapports des médecins coloniaux (figure 1a) et ceux des chercheurs de l’Organisation de Coopération et de Coordination des Grandes Endémies (OCCGE), ainsi que dans celle de l’Institut de Médecine Tropicale du Service de Santé des Armées (IMTSSA) localisé à Marseille au moment de nos travaux.

### Données contemporaines sur la maladie du sommeil

En mars 2011 nous nous sommes rendus à Koudougou au Burkina Faso. Nous avons cherché des informations épidémiologiques contemporaines sur la maladie du sommeil au niveau de la Direction Régionale de la Santé (DRS). Un registre qui présente les cas dépistés dans le district sanitaire de Koudougou de 1971 à 1987 a été retrouvé et consulté (figure 1b).

### Enquéte géographique des trypanosomés dépistés depuis 2000

Grâce aux feuilles individuelles tenues par l’infirmier d’État en charge de la maladie du sommeil à l’hôpital de Koudougou, nous avons pu identifier 13 trypanosomés dépistés depuis 2000 (figure 1c). Nous avons parcouru la région pour retrouver ces trypanosomés et les questionner sur leurs lieux de vie au Burkina Faso et en Côte d’Ivoire. Dix trypanosomés ont pu être retrouvés et interrogés (les trois manquants étaient originaires de districts voisins) (figure 1d). Les coordonnées géographiques des habitations des trypanosomés au Burkina Faso ont été relevées à l’aide du Global Positioning System (GPS Garmin 76cx) et insérées dans un Système d’Information Géographique (SIG, Arcview 3.2) pour la cartographie.

## Résultats

### Koudougou(s) : des villages Ivoiro-Burkinabés poursuivis par la maladie du sommeil

**Figure 2.**
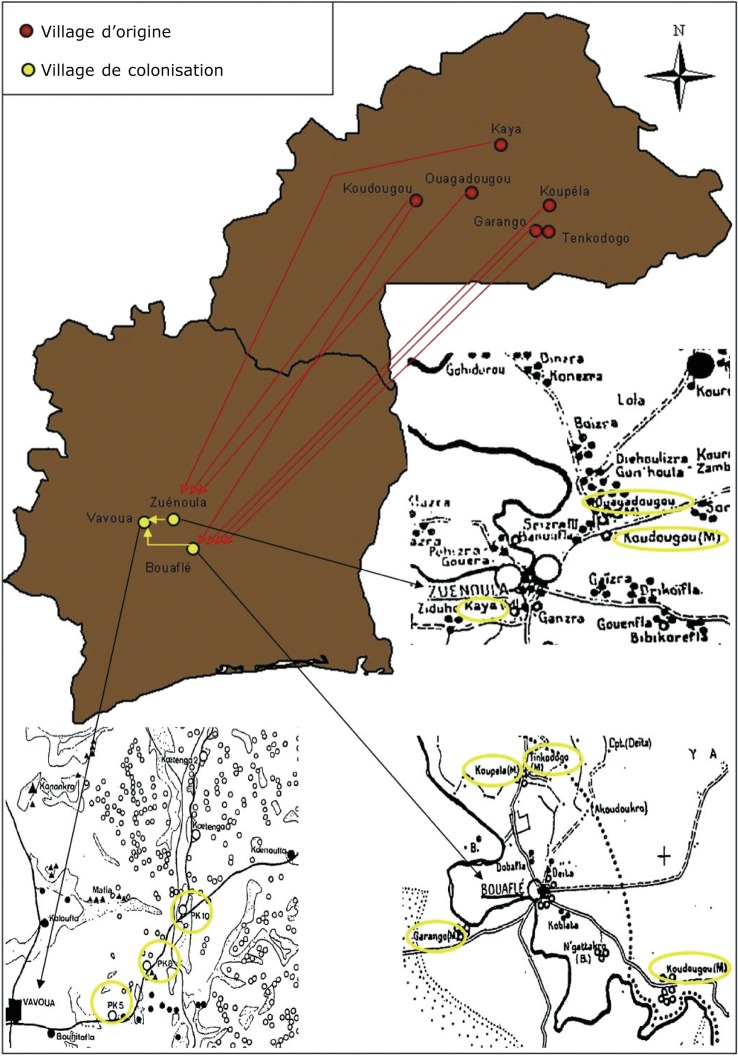
Localisation des villages fondateurs et des villages de colonisation Mossi.

Une des solutions préconisées par l’administration coloniale française pour implanter les populations en zone forestière ivoirienne a été la création de villages de colonisation peuplés par des voltaïques Mossi et Bissa. Dans le cadre de ces initiatives de peuplements, les politiques de séduction (exonération d’impôts, octroi de lopins de terre, administration par des chefs traditionnels) ont permis de recruter la première année (1936) 700 volontaires originaires des cercles voltaïques de Koudougou, Koupéla, Kaya, Ouagadougou (Mossi), ainsi que de Garango et Tenkodogo (Bissa) (figure 2). Ces villages implantés dans le Centre-Ouest ivoirien ont la particularité d’avoir gardé le nom des villages burkinabés d’origine. Ainsi, en Côte d’Ivoire, sept villages étaient créés en pays Gouro : quatre dans la subdivision de Bouaflé (Koudougou, Garango, Tenkodogo, Koupéla) et trois dans la subdivision de Zuénoula (Koudougou, Ouagadougou, Kaya) (Mandé, 1997 ). Parmi ces migrants, se trouvaient de nombreux trypanosomés, dont le nombre variait en fonction du niveau de contamination de la région d’origine. En effet, en Haute-Volta, les cercles de Tenkodogo et Koupéla étaient peu touchés par la maladie du sommeil, contrairement à ceux de Ouagadougou, Kaya, Garango et Koudougou (Domergue-Cloarec, 1986).

Koudougou était sans conteste le cercle le plus touché par la maladie, mais aussi l’un des plus sollicités par la colonie ivoirienne pour l’approvisionnement en main-d’oeuvre, car densément peuplé, et placé en première ligne sur l’axe du chemin de fer Abidjan- Ouagadougou (Capron & Kohler, 1975). En 1933, le commandant du cercle de Koudougou plaide pour une réduction des prestations liées aux travaux forcés, en faisant valoir que chaque recensement permet de constater une diminution de la population. C’est que “*trois mille jeunes gens dans la force de l’âge, les plus aptes aux travaux de culture, sont annuellement enlevés à la terre : un tiers est recruté pour la Côted’Ivoire, le second tiers pour Ouagadougou et le reste pour l’armée*” (Kohler, 1972). C’est dans ce contexte que sévit la maladie du sommeil et les chiffres donnés par Jamot sur le degré de contamination de ce cercle lors du bilan de son action sont éloquents: “*Ajoutons enfin que la visite partielle des Cercles de la Haute- Volta avait permis d’y découvrir au 1er Octobre 1934, 31 854 malades, dont 6 264 à Ouagadougou, 6 877 à Gaoua, 2 104 à Dédougou, 876 à Bobo et 15 733 à Koudougou*” (Jamot, 1935). Autrement dit, la moitié des trypanosomés dépistés par Jamot en Haute-Volta provenait du cercle de Koudougou-BF, principal pourvoyeur de main-d’oeuvre pour la Côte d’Ivoire.

Avec l’organisation de la lutte à travers la création du Service Général Autonome de la Maladie du Sommeil (SGAMS) en 1939, la situation épidémique particulière de Koudougou-BF incita à l’intégrer dans le secteur spécial n° 3 de Koudougou-Mossi (Muraz, 1943). Ce secteur avait la caractéristique d’avoir une surpopulation (164 000 habitants) et une haute infestation générale (5 %), pouvant atteindre 15 % dans certains cantons tels que Lallé et Sourgou. Ce secteur rencontrait d’énormes problèmes pour traiter les malades dépistés, car dans ce cercle à forte émigration de main-d’oeuvre, la difficulté était grande pour retrouver les malades partis soit en Gold Coast (actuel Ghana), soit en Basse Côte d’Ivoire (Scott, 1965).

En zone forestière ivoirienne, l’intensité du contact entre les migrants Mossi (dont certains infectés) et la mouche tsé-tsé *Glossina palpalis palpalis*, espèce éclectique dont les densités ont été favorisées par les débuts de la déforestation, a très probablement permis à la THA de se développer autour de ces villages de colonisation. Jusqu’en 1953, ces villages ont constitué un gros problème de THA dans le secteur n° 14 de Daloa, un des plus contaminés de la zone forestière ivoirienne (Domergue-Cloarec, 1986). C’était le plus méridional de tous les secteurs spéciaux qui comprenait à sa création les subdivisions de Daloa, Vavoua, Zuénoula, Bouaflé, Oumé, Sinfra et Issia, soit environ 205 000 habitants. En 1943, les prévalences tournaient autour de 0,35 % dans le secteur, sauf dans les villages de colonisation Mossi de Bouaflé (Koudougou, Garango) que l’on jugeait très contaminés. De la même manière, dans la zone de Zuénoula, il est rapporté qu’“*en 1945, entre les deux Bandama, les subdivisions de Zuénoula, Mankono et Oumé étaient peu contaminées à l’exception des îlots Mossi (Koudougou, Ouagadougou, Kaya)*” (Domergue-Cloarec, 1986). Si au fur et à mesure du contrôle de l’endémie en Haute- Volta, les migrations en direction de la Côte d’Ivoire comportaient de moins en moins de trypanosomés, les migrants se faisaient désormais contaminer sur place où la maladie circulait (les malades dépistés étaient tous en première période, signe d’une contamination récente) (Domergue-Cloarec, 1986). Progressivement, ces migrants ont remplacé la forêt située aux alentours de leurs villages par des plantations de café et de cacao, à tel point que l’accès à la terre est progressivement devenu compliqué. Ainsi, de nouveaux fronts pionniers ont été mis en place spontanément, en direction des zones de faible peuplement, comme par exemple à Vavoua, située à environ 30 kilomètres à l’Ouest de Zuénoula (figure 2).

Là encore, le nom de Koudougou apparaît, au niveau de trois villages Mossi dénommés Koudougou PK5, Koudougou PK8 et Koudougou PK10 (ou Koudougoucarrefour). Les premiers migrants burkinabés de ces villages semblent être arrivés entre 1951 et 1954. Ils se seraient d’abord installés à Bouhitafla (village Gouro) et auraient ensuite créé leur propre campement vers 1956 : PK5. Ont ensuite été créés PK8 en 1957 et PK 10 (Koudougou-carrefour) en 1958 (Prady, 1983). D’après Prady (1983), “*Le bassin migratoire en Haute-Volta dont sont originaires les planteurs Mossi de la zone étudiée* (Vavoua) *est étroit : il est centré sur la partie Nord de la sous-préfecture de Koudougou*”. Le village d’origine burkinabé des premiers arrivés fournit par la suite une part importante des immigrants. Les plus anciens de PK5 sont originaires de l’arrondissement de Sabou, notamment du village de Sourgou. À PK8, les fondateurs sont de Lallé (village situé à 20 kilomètres à l’est de Kindi). Le chef de PK 10 est aussi venu de Lallé. Deux régions ivoiriennes jouent un rôle essentiel dans l’apport de colons Mossi dans la zone de Vavoua : Zuénoula (d’où sont venus 45 planteurs de 1950 à 1979) et Bouaflé (dix planteurs sur la même période) (Prady, 1982). La place de Zuénoula est considérable au tout début de l’implantation des colons à Vavoua, car tous les premiers arrivés, et en particulier tous les fondateurs des villages Mossi, sont passés par Zuénoula. 25 ans après l’arrivée du premier colon Mossi en provenance de Zuénoula et 15 ans après la constitution des villages de PK5, PK8 et PK 10 (Koudougoucarrefour), le foyer de THA de Vavoua est découvert.

Dans ce foyer, “*l’ethnie ivoirienne principale est constituée par les Gouro ; nous trouvons également quel ques Baoulé et, surtout, une importante main-d’oeuvre étrangère, essentiellement voltaïque. Certains villages, dits “villages Mossi”, sont formés presque uniquement de travailleurs voltaïques et de leurs familles*” et “*une étude de localisation géographique des nouveaux cas pour 1975 et 1976 montre qu’ils sont, pour la plupart, localisés sur quatre villages le long de la route Vavoua- Trafla : il s’agit des villages de Koudougou-PK5, Koudougou- PK8, Koudougou-carrefour et de Koétinga (autre village Mossi)*” (Duvallet *et al.*, 1977). Après la découverte de ce foyer, une équipe de l’Office de Recherche Scientifique et Technique d’Outre-Mer (ORSTOM, actuel IRD), composée d’entomologistes, de médecins et de géographes de la santé a mis en place une lutte médicale et entomologique (Laveissière *et al.*, 1986). Au total, de 1975 à 1983, 932 malades ont été dépistés dans le foyer de Vavoua, parmi lesquels 804 Mossi et 128 autochtones Gouro. Cette lutte semble avoir été un succès puisque seulement quatre malades ont été ensuite dépistés passivement en 1984 au Projet de Recherches Cliniques sur la Trypanosomiase (PRCT), basé à Daloa.

La difficulté d’accéder aux populations disséminées dans des centaines de campements situés aux fins fonds de la forêt a toujours été un obstacle important à la lutte médicale, tout comme les incessants allers et retours de ces migrants entre la région ivoirienne d’accueil et la région burkinabé d’origine, posant la question du risque de propagation de la maladie vers le Burkina Faso. C’est le cas à Vavoua où Laveissière et Couret constatent que “*à l’heure actuelle, la majorité des manoeuvres trypanosomés ont regagné leurs villages d’origine et ne sont plus que des numéros sur les registres du secteur*” (Laveissière & Couret, 1980). Dès lors, le problème qui commença à se poser était le risque de propagation de la maladie du sommeil de la zone forestière ivoirienne toujours contaminée, vers la savane burkinabé nettement assainie par rapport à la situation qui prévalait 40 ans plus tôt.

### Quand les Koudougoulais de Côte d’Ivoire rentrent à Koudougou au Burkina Faso

**Figure 3.**
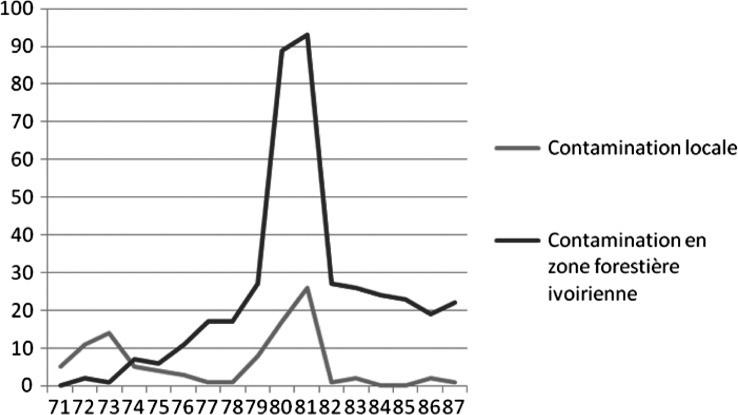
Nombre de trypanosomés dépistés à Koudougou de 1971 à 1987 par zone probable de contamination.

Dans les années 1970–80, pendant que la maladie du sommeil continuait de se répandre en zone forestière ivoirienne, quelques foyers de THA subsistaient au Burkina Faso, comme à Koudougou. L’analyse des données saisies dans le répertoire retrouvé dans les locaux de la DRS de Koudougou montre l’évolution du nombre de cas dépistés par année et en fonction de leur provenance (Burkina Faso/Côte d’Ivoire) pour la période 1971–1987 (figure 3). Dans cet intervalle de temps, 512 trypanosomés ont été dépistés à l’hôpital de Koudougou, il s’agit alors de dépistages passifs. Sur ces 512 trypanosomés, 101 (20 %) ont été identifiés (probablement sur la base de questionnaires) comme contaminés au Burkina Faso et 411 (80 %) en Côte d’Ivoire. La figure 3 montre l’inversion de l’origine géographique des lieux de contamination des trypanosomés dépistés passivement à Koudougou. Avant 1974, la majorité des malades dépistés sont infectés localement dans la région de Koudougou, et ce n’est qu’à partir de 1974 que la plupart des malades dépistés sont contaminés en Côte d’Ivoire. Après 1974, le nombre de cas dépistés localement baisse de manière continue jusqu’en 1979, tandis que le nombre de cas dépistés en provenance de Côte d’Ivoire augmente. En plus de ces phénomènes, apparaissent très clairement deux pics pour les années 1979, 1980, 1981 et 1982, qui correspondent à une augmentation très importante du nombre de trypanosomés, dépistés à la fois en provenance du Burkina Faso et de Côte d’Ivoire.

**Figure 4.**
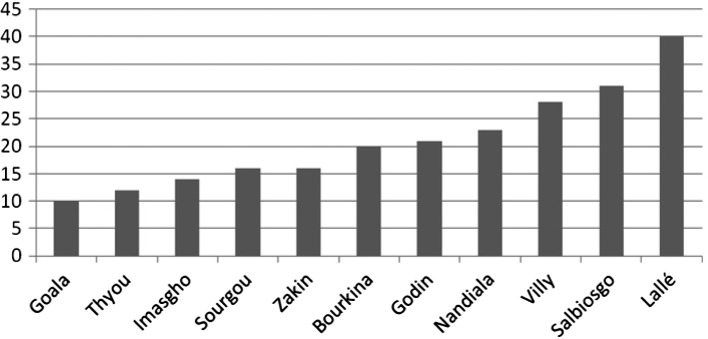
Nombre de trypanosomés dépistés passivement à l’hôpital de Koudougou (Burkina Faso) sur la période 1971–1987 en fonction du village d’origine.

À la fin des années 1980, le nombre de trypanosomés dépistés passivement à Koudougou a fortement diminué (23 cas en 1987), avec deux cas identifiés comme contamination locale. La transmission locale du trypanosome semble donc sur le point de disparaître. Les villages burkinabés dont sont originaires les malades dépistés sur la même période montrent que c’est du village de Lallé, dont sont issus les chefs fondateurs des villages Mossi de Zuénoula et de Vavoua, que proviennent la plupart des cas (figure 4). Étant donné que la quasi-totalité des cas sont issus d’une contamination en Côte d’Ivoire, il est fort probable que ces trypanosomés soient revenus infectés des zones de Bouaflé, Zuénoula et surtout Vavoua. Cette même analyse n’est pas possible pour les villages ivoiriens de provenance puisque sur le registre est uniquement notée la mention CI (Côte d’Ivoire). De 1987 jusqu’à 2000, aucune information épidémiologique n’a pu être retrouvée pour le district sanitaire de Koudougou-BF. Aujourd’hui, les trypanosomés dépistés à Koudougou sont toujours des migrants de retour de Côte d’Ivoire, mais qui sont-ils et d’où viennent-ils ?

### Les Trypanosomés dépistés passivement depuis 2000 à Koudougou-BF

**Figure 5.**
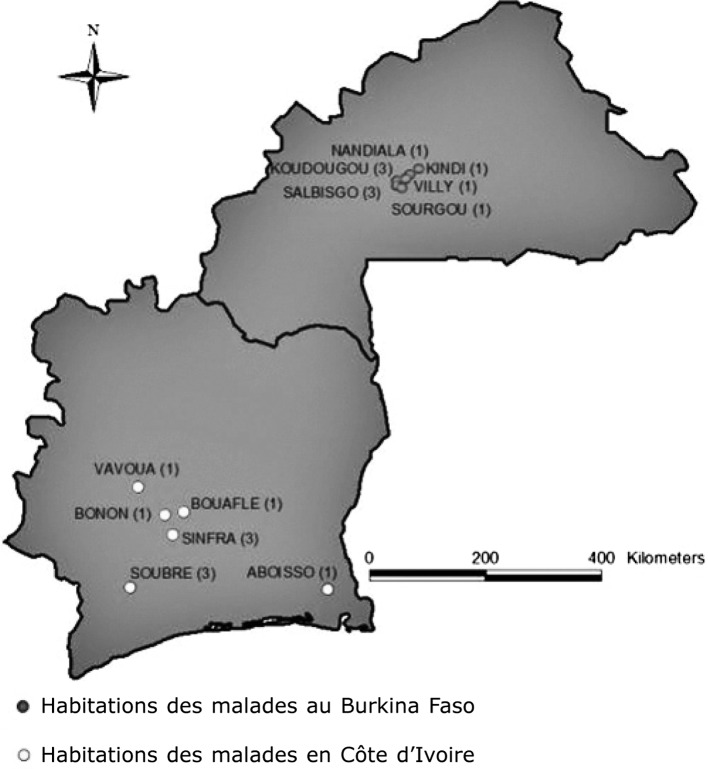
Lieux d’habitations des trypanosomés dépistés entre 2000 et 2010 à Koudougou au Burkina Faso et zones de provenance ivoirienne.

Nos recherches ont permis de retrouver et d’enquêter dix trypanosomés dépistés depuis 2000 à Koudougou- BF (figure 5). Tous les cas sont des Mossi originaires de la zone de Koudougou-BF. Sans surprise, neuf cas proviennent du Centre-Ouest (Bonon, Sinfra, Vavoua, Soubré) et un du Sud-Est (Aboisso) de la Côte d’Ivoire. Cette distribution confirme bien la distribution spatiale habituelle de la maladie du sommeil de ces 20 dernières années en Côte d’Ivoire (Djé *et al.*, 2002; Kaba *et al.*, 2006). L’identification des lieux précis de provenance de ces cas donne des informations capitales en termes d’orientation géographique des prospections médicales pour la Côte d’Ivoire. Les trois malades dépistés en 2010 proviennent du triangle Bonon-Sinfra-Bouaflé et plus précisément des villages de Kangréta, Loafla et Sourgou. Trois trypanosomés dépistés en 2004, 2005 et 2010 proviennent de la zone de Sinfra (deux viennent de Loafla et un de Bourkoro). Ces villages ivoiriens, en particulier les deux derniers, constituent donc une priorité en termes de surveillance épidémiologique. Les lieux de résidence à Koudougou-BF et les villages alentours ne constituent pas un risque de réactivation du cycle de transmission, car les glossines sont aujourd’hui absentes de cette région.

## Discussion-Conclusion

Les mouvements historiques de populations du Burkina Faso vers la Côte d’Ivoire ont permis la propagation de la maladie du sommeil en forêt ivoirienne et participé ainsi au développement des foyers de THA dans ce pays. Certains îlots de peuplement ont constitué en forêt ivoirienne les foyers de THA les plus virulents, comme ceux de Koudougou-CI (Domergue-Cloarec, 1986). Ainsi, de Koudougou-Bouaflé à Koudougou-Zuénoula en passant par Koudougou-Vavoua, les villages de Koudougou et les centaines de campements satellites qui les ont accompagnés en périphéries, pour le développement des plantations de café et de cacao, ont constitué la piste principale de la maladie du sommeil en forêt ivoirienne. Cette piste, les médecins et chercheurs en charge de la lutte n’ont pas pu la suivre assez rapidement pour stopper la progression de la maladie, et pour cause, ce sont des milliers de migrants particulièrement mobiles qui l’ont constituée. En effet, les migrants burkinabés (parmi lesquels des trypanosomés) ont sillonné la forêt ivoirienne en vendant leur force de travail dans une multitude de plantations jusqu’à ce qu’ils trouvent une parcelle de terre à acquérir (Boni, 1978). Cette mobilité intraforestière s’est déroulée à une époque où la forêt était encore propice aux fortes densités de glossines et surtout constituait une mosaïque paysagère particulièrement favorable au contact homme/vecteur (Laveissière *et al.*, 1986). Dans ce contexte, la maladie du sommeil trouvait donc toutes les conditions nécessaires à sa transmission et à sa diffusion, d’autant plus que la stérilisation du réservoir de parasites était rendue difficile du fait (*i*) de l’inaccessibilité des migrants disséminés dans une multitude de hameaux répartis aux fins fonds de la forêt, (*ii*) de leur mobilité importante dans la zone et (*iii*) de leurs incessants allers et retours entre la Côte d’Ivoire et le Burkina Faso, leur pays d’origine.

Aujourd’hui, les glossines ont disparu de la zone de Koudougou au Burkina Faso (Courtin *et al.*, 2010a). Cette réalité écarte l’hypothèse de l’existence d’une transmission locale actuelle du trypanosome. L’analyse des lieux de contamination des malades dépistés passivement à Koudougou sur la période 1971–1987 montre que la contamination locale commençait à diminuer fortement à partir des années 1970, au profit de la contamination en Côte d’Ivoire et qu’elle était sur le point de disparaître en 1987 avec seulement deux cas contaminés localement. La baisse drastique du nombre de cas infectés localement à partir de 1974 et l’augmentation continuelle du nombre de cas dépistés en provenance de Côte d’Ivoire à partir de 1974 pourraient très bien refléter l’impact de la sécheresse des années 1972–1973 qui a :

1. accentué la pression anthropique (cultures) au niveau des forêts-galeries et modifié les conditions de température et d’humidité aux abords des cours d’eau, conditions déjà extrêmes pour les mouches avant ces épisodes de sécheresse dans la zone de Gogho situés à une vingtaine de kilomètres au sud-est de Koudougou (Challier & Dedewanou, 1967). Il s’en est suivi une rétraction de l’aire de distribution des glossines qui ont subi ces évolutions climatiques associées à une augmentation des densités de population humaines (Laveissière *et al.*, 1977) ;
2. encouragé les populations (pasteurs, agriculteurs) à migrer vers le Sud plus humide et notamment en forêt ivoirienne, comme par exemple dans la région de Vavoua, foyer de maladie du sommeil particulièrement actif à ce moment-là (Duvallet *et al.*, 1977 ; Prady, 1982).

L’augmentation du nombre de cas dépistés passivement à Koudougou-BF de 1979 à 1982 est parfaitement corrélée à la lutte médicale menée dans le foyer de Vavoua en Côte d’Ivoire (où les Mossi sont originaires de Koudougou) de 1976 à 1983. Les malades dépistés à Vavoua en Côte d’Ivoire ont probablement préféré se faire soigner “au pays”, c’est-à-dire à l’hôpital de Koudougou.

Depuis 2000, des trypanosomés originaires de la région de Koudougou continuent d’être dépistés à l’hôpital de Koudougou. Si dans cette région, ce retour de trypanosomés ne pose plus le problème de réactivation du cycle de transmission (du fait de la disparition des glossines), il n’en va pas de même pour le Sud-Ouest du Burkina Faso, où les glossines sont encore présentes, parfois en grandes densités (Rayaisse *et al.*, 2009). Cette région du Burkina Faso très convoitée par les migrants Mossi à la recherche d’une terre à cultiver est aussi concernée par les importants mouvements de populations avec la Côte d’Ivoire (Courtin *et al.*, 2010b). La réintroduction du parasite est donc potentielle, comme en témoigne un trypanosomé provenant de Côte d’Ivoire dépisté en 2011 dans la zone de Diébougou (voir Kambiré *et al.*, 2012 de ce même volume de *Parasite*). Des facteurs indispensables à la réémergence d’un foyer de maladie du sommeil au Burkina Faso sont encore présents dans certaines régions, mais la probabilité de rencontre simultanée des trois acteurs du cycle dans un même lieu et au même moment reste heureusement faible. En effet, le nombre de trypanosomés séjournant dans ces zones à risque est relativement peu élevé, et l’intensité du contact entre l’homme et la glossine a fortement diminué ces dernières années. Premièrement parce que les densités de glossines ont considérablement diminué, et deuxièmement parce que le développement des aménagements hydrauliques rend l’homme moins fréquent aux abords des cours d’eau, lieu de rencontre privilégié avec la glossine en zone de savane (Laveissière *et al.*, 2000; Courtin *et al.*, 2009). Néanmoins, ce risque existe et il doit être pris en compte dans les stratégies de surveillance du Programme National de Lutte contre la THA burkinabé.

D’autre part, notre travail pose aussi des questions cruciales par rapport à la situation de la maladie en Côte d’Ivoire. En effet, ce pays sort d’une décennie d’instabilité politique profonde (2002–2011), durant laquelle les équipes de santé n’ont pas pu jouer pleinement leur rôle de surveillance (Kaba *et al.*, 2006). Sans compter les déplacements de populations favorables à la propagation et les difficultés pour les malades étrangers d’accéder aux soins. Cette situation nécessite de faire le point sur la situation épidémiologique de la THA en Côte d’Ivoire.

L’origine géographique ivoirienne des trypanosomés dépistés à Koudougou est un élément primordial pour l’orientation de la surveillance médicale en Côte d’Ivoire. L’évidente corrélation entre les itinéraires géographiques suivis par les personnes originaires de Koudougou en Côte d’Ivoire et le développement des foyers de maladie du sommeil fait de ce facteur un critère essentiel à prendre en compte. Nous ne détaillerons pas plus ces deux derniers aspects, car ils sont largement discutés dans l’article de Kambiré *et al.* (2012) de ce même volume, dont l’objectif est d’optimiser les stratégies de surveillance en tenant compte du contexte épidémiologique actuel au Burkina Faso et en Côte d’Ivoire.
